# Extension of GWAS results for lipid-related phenotypes to extreme obesity using electronic health record (EHR) data and the Metabochip

**DOI:** 10.3389/fgene.2014.00222

**Published:** 2014-08-05

**Authors:** Ankita Parihar, G. Craig Wood, Xin Chu, Qunjan Jin, George Argyropoulos, Christopher D. Still, Alan R. Shuldiner, Braxton D. Mitchell, Glenn S. Gerhard

**Affiliations:** ^1^Department of Medicine and Program for Personalized and Genomic Medicine, University of Maryland School of MedicineBaltimore, MD, USA; ^2^Geisinger Clinic, Geisinger Obesity InstituteDanville, PA, USA; ^3^Department of Pathology and Laboratory Medicine, Department of Biochemistry and Molecular Biology, Institute for Personalized Medicine, Pennsylvania State University College of MedicineHershey, PA, USA; ^4^Geriatric Research and Education Clinical Center, Veterans Administration Medical CenterBaltimore, MD, USA

**Keywords:** GWAS, lipids, obesity, EHR

## Abstract

A variety of health-related data are commonly deposited into electronic health records (EHRs), including laboratory, diagnostic, and medication information. The digital nature of EHR data facilitates efficient extraction of these data for research studies, including genome-wide association studies (GWAS). Previous GWAS have identified numerous SNPs associated with variation in total cholesterol (TC), low-density lipoprotein cholesterol (LDL-C), high-density lipoprotein cholesterol (HDL-C), and triglycerides (TG). These findings have led to the development of specialized genotyping platforms that can be used for fine-mapping and replication in other populations. We have combined the efficiency of EHR data and the economic advantages of the Illumina Metabochip, a custom designed SNP chip targeted to traits related to coronary artery disease, myocardial infarction, and type 2 diabetes, to conduct an array-wide analysis of lipid traits in a population with extreme obesity. Our analyses identified associations with 12 of 21 previously identified lipid-associated SNPs with effect sizes similar to prior results. Association analysis using several approaches to account for lipid-lowering medication use resulted in fewer and less strongly associated SNPs. The availability of phenotype data from the EHR and the economic efficiency of the specialized Metabochip can be exploited to conduct multi-faceted genetic association analyses.

## Introduction

Genome-wide association studies (GWAS) have been highly successful at identifying SNPs associated with a wide variety of phenotypes, including lipid disorders, although such studies require very large sample sizes (Willer et al., 2013). This limits their utility because of economic considerations and the need to acquire phenotype data from across diverse sources. These limitations can be minimized using data obtained from electronic health records (EHRs), which can be an efficient means to obtain robust and extant phenotype data (Pathak et al., 2012) from potentially a large number of individuals, including for metabolic traits (Wood et al., 2012) and genetics studies (Wood et al., 2008). Furthermore, this approach provides the opportunity to assess the relevance of genetic associations in real-world patient populations with selected phenotypic characteristics such as extreme obesity. Because lipid screening is part of standard of care testing and body weights are often measured, these data are commonly present in EHRs. The electronic nature of EHR data facilitates efficient extraction for research studies (Prokosch and Ganslandt, 2009). However, the accuracy of EHR-based data depends upon how the data were obtained and entered and how it was extracted. Certain portions of the EHR are more standardized, such as laboratory measures. Other data, such as medications, may not be as straight-forward because of the complexity of coding for medication use.

Large meta-analyses of GWAS have identified numerous genetic loci associated with variation in lipid phenotypes, including 39 loci for total cholesterol (TC), 22 loci for low-density lipoprotein cholesterol (LDL-C), 31 loci for high-density lipoprotein cholesterol (HDL-C), and 16 loci for triglycerides (TG) (Van Dongen et al., 2013) as well as body mass index (BMI) (Sandholt et al., 2012). These loci are estimated to underlie about one-quarter to one-third of the genetic basis for these traits, a result that has motivated the search for additional loci through even larger GWAS studies (Willer et al., 2013). Few GWAS have been conducted in populations with extreme obesity (Sarzynski et al., 2011; Rinella et al., 2013), which may differ significantly from the large GWAS population-based samples in prevalence of co-morbidities such as dyslipidemia and use of corresponding lipid lowering medications.

Results from GWAS data have led to the development of specialized platforms designed to identify additional genetic loci as follow-up to initial analyses and to allow for finer genetic mapping of previously identified loci. An economical genotyping platform, the Illumina Metabochip, was custom designed for analysis of traits related to coronary artery disease and type 2 diabetes (Voight et al., 2012). As a proof-of-principle use of EHR data, we genotyped DNA from a cohort of individuals with extreme obesity, ascertained on the basis of undergoing bariatric surgery and on whom a rich database of EHR-derived phenotype data were available (Still et al., 2013), using the Metabochip to evaluate associations with lipid traits. The presence of SNPs on the Metabochip residing in loci known to be previously associated with blood lipid levels enabled extension of findings to the context of extreme obesity. The use of EHR data and the Metabochip platform thus provided an effective strategy to test the relevance of lipid trait GWAS findings in this patient population.

## Materials and methods

### Study participants, EHR source data, and collection of blood samples

Study participants were patients treated in the Geisinger Clinic Center for Nutrition and Weight Management who met clinical inclusion and exclusion criteria for bariatric surgery and were prospectively recruited into a research program on obesity from 2004 to 2012. The Geisinger Health System is an integrated health care delivery system that serves residents in central and northeastern Pennsylvania that includes the Geisinger Clinic, a network of 37 community-based primary care practices that provide care to over 400,000 patients. All sites have used the EpicCare™ EHR since 2001 (Allen-Ramey et al., 2013).

Data used for this study were obtained from an obesity database based on the EHR as previously described (Wood et al., 2012). Source data included patient demographics, clinical measures, problem list based on ICD-9 codes, medical history, medication history, and lab results. Blood drawn for lipid measurements and DNA isolation was obtained as part of a standard of care phlebotomy performed during the pre-surgery period, which consisted of a 6–12 month program during which a comprehensive medical history was obtained, a physical exam conducted, body weight, waist circumference, and height measured, and disease-specific, standard of care laboratory tests obtained, including fasting TC, HDL-C, LDL-C, and TG. Clinical data were recorded in the EHR. Blood for DNA isolation was transported to the research laboratory for processing and storage. Genomic DNA was isolated from patient whole blood samples as previously described (Chu et al., 2008), arrayed into microplates, and transported to the University of Maryland Translational Genomics Laboratory for Metabochip genotyping. The research was approved by the Geisinger Clinic and Penn State Hershey Institutional Review Boards and all participants provided written informed consent.

### Genotyping and genotype cleaning

A total of 1851 samples was selected for genotyping using the Illumina Metabochip. The Metabochip array consists of ~200,000 SNPs that include: (1) “replication” SNPs corresponding to validated associations; (2) a set of 63,450 SNPs that were the most significantly associated with over 20 traits related to coronary artery disease or T2D, including lipids, (3) SNPs previously associated with BMI and waist circumference, as well as 122,241 SNPs to fine-map these loci; and (4) 16,992 other SNPs selected for a variety of reasons, including those that reached genome-wide significance in any GWAS (Voight et al., 2012; Shah et al., 2013). Genotyping of the Metabochip was performed as per the manufacturer's protocol. A total of 196,725 were polymorphic. Samples that had call rates across all SNPs of <95% were removed, leaving a total of 1827 samples (Supplementary Table 1). Eight of the 1827 samples were excluded due to missing phenotype data. After excluding samples discordant for reported and genetically determined sex, unresolvable duplicates, and samples related to another sample (Supplementary Methods), the remaining number of subjects available for the analysis set was 1686 (Supplementary Table 1).

A series of analyses were also conducted to identify potentially problematic SNPs. Starting with the 196,725 polymorphic SNPs, we identified a total of 6279 problematic SNPs (Supplementary Methods) that were excluded, with the final cleaned dataset consisting of a total of 190,446 SNPs of which 63,134 SNPs had minor allele frequencies <0.01.

### Association analysis

Statistical association testing between individual SNPs and lipid phenotypes was conducted under an additive model by regressing the genotype score (coded as number of copies of the reference allele) against the outcome lipid variable. Age and sex were included in the model as covariates. Further analyses addressed the issue of use of lipid-lowering agents (see Results). Our initial aim was to assess associations with 21 SNPs present on the Metabochip previously associated with lipid levels in prior meta-analysis of GWAS results (Kathiresan et al., 2008). For these analyses, we regarded a *p*-value of 0.0024 (0.05/21) to be statistically significant. We additionally assessed associations of 21 SNPs previously associated with body mass index and waist circumference (Willer et al., 2009; Speliotes et al., 2010; Sandholt et al., 2012), regarding a *p*-value of 0.002 (0.05/21) to be statistically significant.

We performed a secondary analysis to assess associations of all Metabochip SNPs with lipid and body weight traits in which we adjusted for the total number of SNPs tested, defining the significance cut-off as *p* < 2.6 × 10^−7^ after Bonferroni's correction (*p* = 0.05/190,446). We estimated that our final sample size of 1686 individuals provided 80% power to detect SNPs explaining 2–2.5% of the variation in lipid or BMI levels at this significance level.

## Results

### Cohort characteristics

The demographic, anthropometric, and lipid profiles of the population (Table 1) were characteristic of a bariatric surgery cohort (Wood et al., 2012). Over 99% of the population was Caucasian/European ancestry. Just under 46% of study subjects reported taking one or more lipid-lowering medications, the majority taking statins (Supplementary Table 2). The percent of patients with a diagnosis of hypertension was 48.7%. The diagnosis of type 2 diabetes was 35.2% (Supplementary Table 2), which was reflected in the concomitant use of diabetes medications (Supplementary Table 2).

**Table 1:** **Demographic and laboratory data**.

Trait	Female (*n* = 1365)		Male (*n* = 321)		Total (*n* = 1686)	
	Mean	Stdev	Mean	Stdev	Mean	Stdev
BMI (kg/m^2^)	46.5	8.1	48.6	9.1	46.9	8.3
WaistCir (inches)	50.0	12.5	56.8	15.1	51.3	13.3
TG (mg/dl)	162.7	106.2	190.6	145.5	168.0	115.0
TC (mg/dl)	184.8	46.7	168.4	51.1	181.8	48.0
HDL-C (mg/dl)	46.9	13.7	38.4	12.6	45.3	13.9
LDL (mg/dl)	104.3	38.4	88.6	42.5	101.4	39.7
TC/HDL-C	3.8	2.1	4.1	2.7	3.9	2.2

### Association of SNPs at known lipid loci with lipid levels

Results from association testing of previously identified lipid loci (Kathiresan et al., 2008) are shown in Table 2. Of the 21 lipid-associated SNPs tested, 12 were nominally associated with one or more lipid traits at a *p* < 0.05, including 3 that remained significant following adjustment for multiple comparisons (*p* < 0.002). The loci marked by these three SNPs included *GCKR* (associated with TG levels at 5.3 × 10^−4^), *LPL* (associated with HDL-C levels at 1.4 × 10^−5^), and *CETP* (associated with HDL-C levels at 4.1 × 10^−11^). The directions of the observed effects for all of the 12 SNPs nominally or significantly associated with lipid levels were directionally consistent with those previously reported.

**Table 2:** **Associations of SNPs at known lipid-associated loci with lipid traits**.

Trait	SNP	CHR	CHR position (HG18)	GENE	Ref. allele	Loci previously associated with lipid levels through GWAS from Kathiresan et al. (2008)			All Subjects (*n* = 1686)			Exclude subjects taking lipid-lowering medications (*n* = 945)		Include medication use as a covariate (*n* = 1686)		Include ONLY subjects taking lipid-lowering medications (*n* = 741)	
						Allele freq	Beta^†^	*P*-value	Allele freq	Beta^†^	*P*-value	Beta^†^	*P*-value	Beta^†^	*P*-value	Beta^†^	*P*-value
LDL	rs11206510	1	55,268,627	PCSK9	G	0.19	−0.09	4.00	0.18	−3.32	0.030	−2.51	0.225	−3.38	0.028	−2.70	0.311
								E-08									
HDL	rs4846914	1	228,000,000	GALNT2	G	0.40	−0.05	4.00	0.40	−0.04	0.923	0.35	0.617	−0.05	0.905	−0.33	0.564
								E-08									
TG	rs7557067	2	21,061,717	APOB	G	0.22	−0.08	9.00	0.25	−0.04	0.025	−0.06	0.057	−0.05	0.022	0.00	0.809
								E-12									
LDL	rs515135	2	21,139,562	APOB	A	0.20	−0.16	5.00	0.19	−3.74	0.013	−5.26	0.013	−3.88	0.010	−1.02	0.702
								E-09									
TG	rs1260326	2	27,584,444	GCKR	A	0.45	0.12	2.00	0.43	0.06	5.30	0.07	0.010	0.06	7.60	0.02	0.101
								E-31			E-04				E-04		
LDL	rs6544713	2	43,927,385	ABCG8	A	0.32	0.15	2.00	0.31	3.00	0.019	0.33	0.853	3.12	0.015	3.23	0.145
								E-20									
TG	rs714052	7	72,502,805	MLXIPL	G	0.12	−0.16	3.00	0.11	0.00	0.888	−0.03	0.517	−0.01	0.791	0.02	0.266
								E-15									
TG	rs7819412	8	11,082,571	XKR6	A	0.48	−0.04	3.00	0.48	0.00	0.931	0.02	0.526	0.01	0.895	0.00	0.805
				AMAC1L2				E-08									
TG	rs12678919	8	19,888,502	LPL	G	0.10	0.23	2.00	0.10	−0.08	0.006	−0.10	0.021	−0.08	0.007	−0.02	0.166
								E-41									
HDL	rs12678919	8	19,888,502	LPL	G	0.10	0.23	2.00	0.10	2.78	1.37	2.74	0.012	2.75	1.59	0.36	0.714
								E-34			E-05				E-05		
TG	rs2954029	8	126,490,972	TRIB1	T	0.44	−0.11	3.00	0.47	−0.04	0.011	−0.03	0.226	−0.04	0.022	−0.03	0.164
								E-19									
HDL	rs1883025	9	107,000,000	ABCA1	A	0.26	−0.08	1.00	0.27	−0.45	0.298	0.16	0.837	−0.43	0.311	−0.15	0.826
								E-09									
TG	rs174547	11	61,327,359	FADS1	G	0.33	−0.09	2.00	0.32	0.03	0.165	0.02	0.541	0.03	0.110	0.01	0.088
				FADS2				E-14									
				FADS3													
HDL	rs174547	11	61,327,359	FADS1	G	0.33	−0.09	2.00	0.32	−0.73	0.069	−0.87	0.202	−0.77	0.053	−1.05	0.543
				FADS2				E-12									
				FADS3													
LDL	rs2650000	12	121388962	HNF1A	A	0.36	0.07	2.00	0.37	1.84	0.127	0.39	0.811	1.89	0.117	1.73	0.425
								E-08									
HDL	rs10468017	15	56,465,804	LIPC	A	0.30	0.1	0.008	0.28	1.10	0.097	0.75	0.293	1.09	0.010	0.67	0.302
HDL	rs173539	16	55,545,545	CETP	A	0.32	0.25	4.00	0.32	2.66	4.09	3.19	3.15	2.67	2.85	0.74	0.242
								E-75			E-11		E-06		E-11		
HDL	rs4939883	18	45,421,212	LIPG	A	0.17	−0.14	7.00	0.17	−1.42	0.005	−0.43	0.647	−1.47	0.004	−2.33	0.003
								E-15									
HDL	rs2967605	19	8,375,738	ANG	A	0.16	−0.12	1.00	0.18	0.34	0.493	0.58	0.479	0.19	0.700	0.87	0.251
								E-08									
LDL	rs6511720	19	11,063,306	LDLR	A	0.10	−0.26	2.00	0.12	−5.03	0.005	−5.20	0.026	−5.27	0.003	−7.74	0.017
								E-26									
LDL	rs10401969	19	19,268,718	NCAN	G	0.06	−0.05	2.00	0.07	−1.64	0.488	0.58	0.856	−1.87	0.431	−9.89	0.030
				E-08				CILP2									
TG	rs17216525	19	19,523,220	NCAN	A	0.07	−0.11	4.00	0.07	−0.07	0.031	−0.08	0.123	−0.06	0.086	−0.04	0.198
				CILP2				E-11									
				PBX4													
TG	rs7679	20	44,009,909	PLTP	G	0.19	−0.07	7.00	0.17	0.05	0.044	0.08	0.026	0.06	0.023	0.00	0.902
								E-11									
HDL	rs7679	20	44,009,909	PLTP	G	0.19	−0.07	4.00	0.17	−0.93	0.060	−1.25	0.157	−1.00	0.043	−0.06	0.934
								E-09									

^*^SNPs at known lipid-associated loci from Kathiresan et al. (2008).

† Effect on lipid levels (expressed in SD units) associated with each copy of the reference allele.

### Adjustment for lipid-lowering medications

A significant proportion (46%) of subjects in this cohort were being treated with lipid-lowering medications. Medication use was not associated with levels of LDL-C (beta = −2.74; *p* = 0.12) or TC (beta = 2.70; *p* = 0.19), but was significantly associated with levels of HDL-C (beta = −2.78; *p* = 1 × 10^−6^) and TG (beta = 0.22; *p* = 1 × 10^−17^). The observed values TC, LDL-C, and TG levels would likely have been higher, and HDL-C levels lower, had they not been taking lipid-lowering medications. We therefore considered three additional analytic approaches to accommodate the effect of the medications. Our first approach was to repeat the association analyses after removing all subjects on lipid-lowering medications (final *n* = 945). Our second approach was to include use of lipid-lowering medications as a covariate (medication user vs. non-user) in the regression model (final *n* = 1686). Our final approach was to restrict analysis to subjects taking lipid-lowering medications (final *n* = 741).Results of the association analyses of SNPs at known lipid loci using all three approaches to address the use of lipid-lowering medications are shown in Table 2. Results obtained from analysis restricted to subjects not taking lipid-lowering medications were generally consistent with those obtained from the initial analysis of the entire cohort. With only a few exceptions (e.g., rs6544713 near ABCG8), the effect sizes at most loci tended to be of the same magnitude, although the *p*-values tended to be less significant in the sample with medication-users removed, consistent with a smaller sample size. The same trend, i.e., comparable effect sizes but lower statistical significance, was also observed when analyses were restricted to subjects taking lipid-lowering medications. Inclusion of medication use as a covariate in the model had virtually no effect on the genotype-lipid phenotype association at any of the tested SNPs.

Array-wide association analysis of lipid levels was also carried out using the same three approaches to evaluate the impact of lipid-lowering medication use. Manhattan plots for these results are shown in Supplementary Figures 3–5. In these analyses, we detected the association of HDL-C with the *CETP* locus at array-wide significance thresholds in subjects not taking lipid-lowering medications and with medication use as a covariate, but not in the subgroup taking lipid-lowering medications (Supplementary Table 4). A similar result was obtained for association of LDL-C with the *APOE* locus. The association of TG with the *APOA1-APOA3-APOA4-APOA5* locus was detected only when using medication use as a covariate.

Association of SNPs at known BMI and waist circumference loci For BMI and waist circumference, no SNP achieved a *p*-value of less than 0.002 (Supplementary Table 5).

### Array-wide association analysis

Following analysis of the candidate SNPs, association analysis was undertaken for all SNPs on the array using an additive genetic model for 7 phenotypes; BMI, waist circumference, TC, LDL-C, HDL-C, TG, and TC/HDL-C ratio. For BMI and waist circumference, no SNP achieved a *p*-value of less than 1 × 10^−6^(Supplementary Figures 1 and 2).

**Figure 1:**
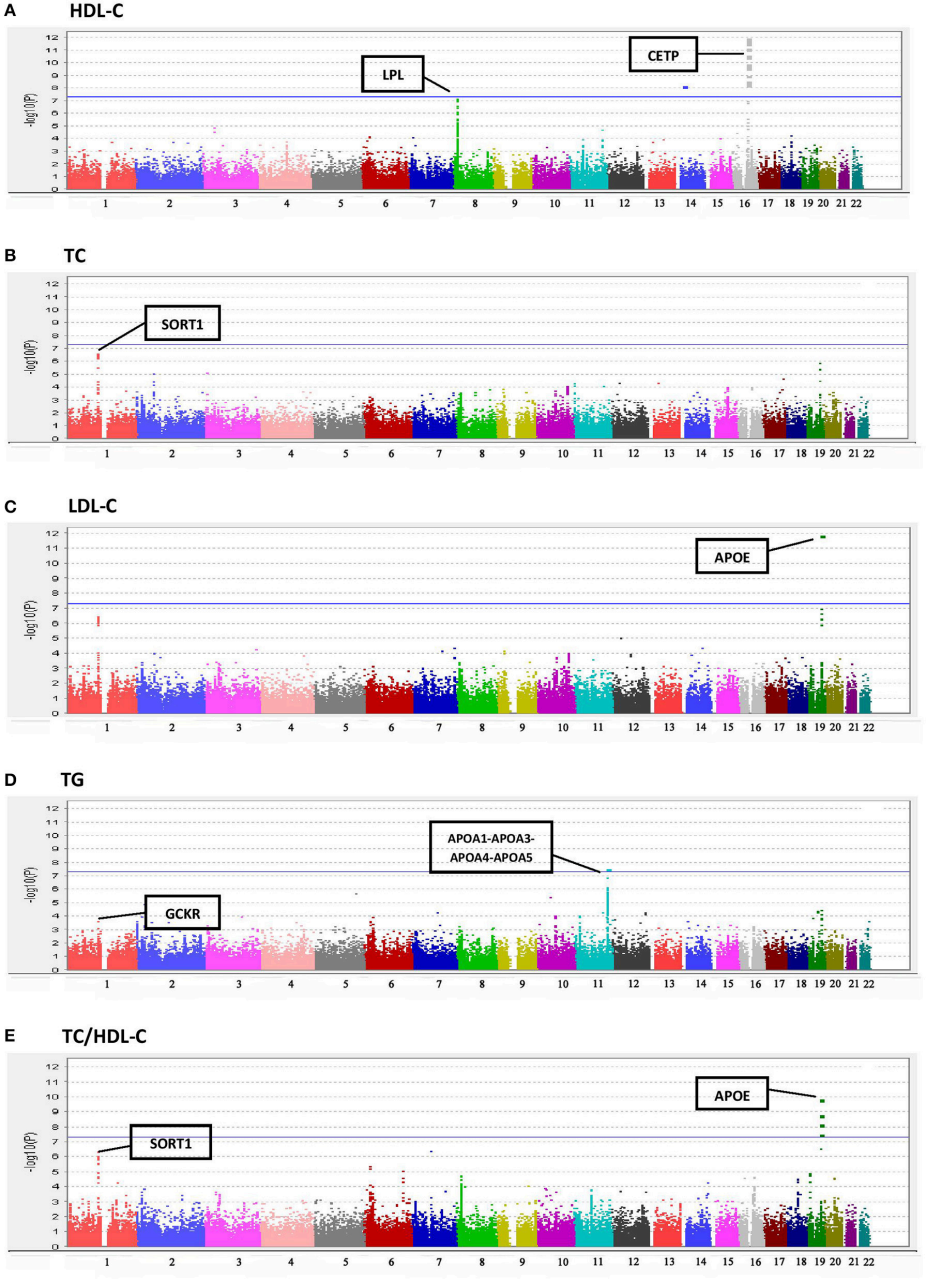
**Manhattan plots for associations of TC, HDL-C, LDL-C, TG, and TC/HDL-C with 190,446 SNPS from the Metabochip. (A)** HDL-C. **(B)** TC. **(C)** LDL-C. **(D)** TG. **(E)** TC/HDL-C.

**Figure 2:**
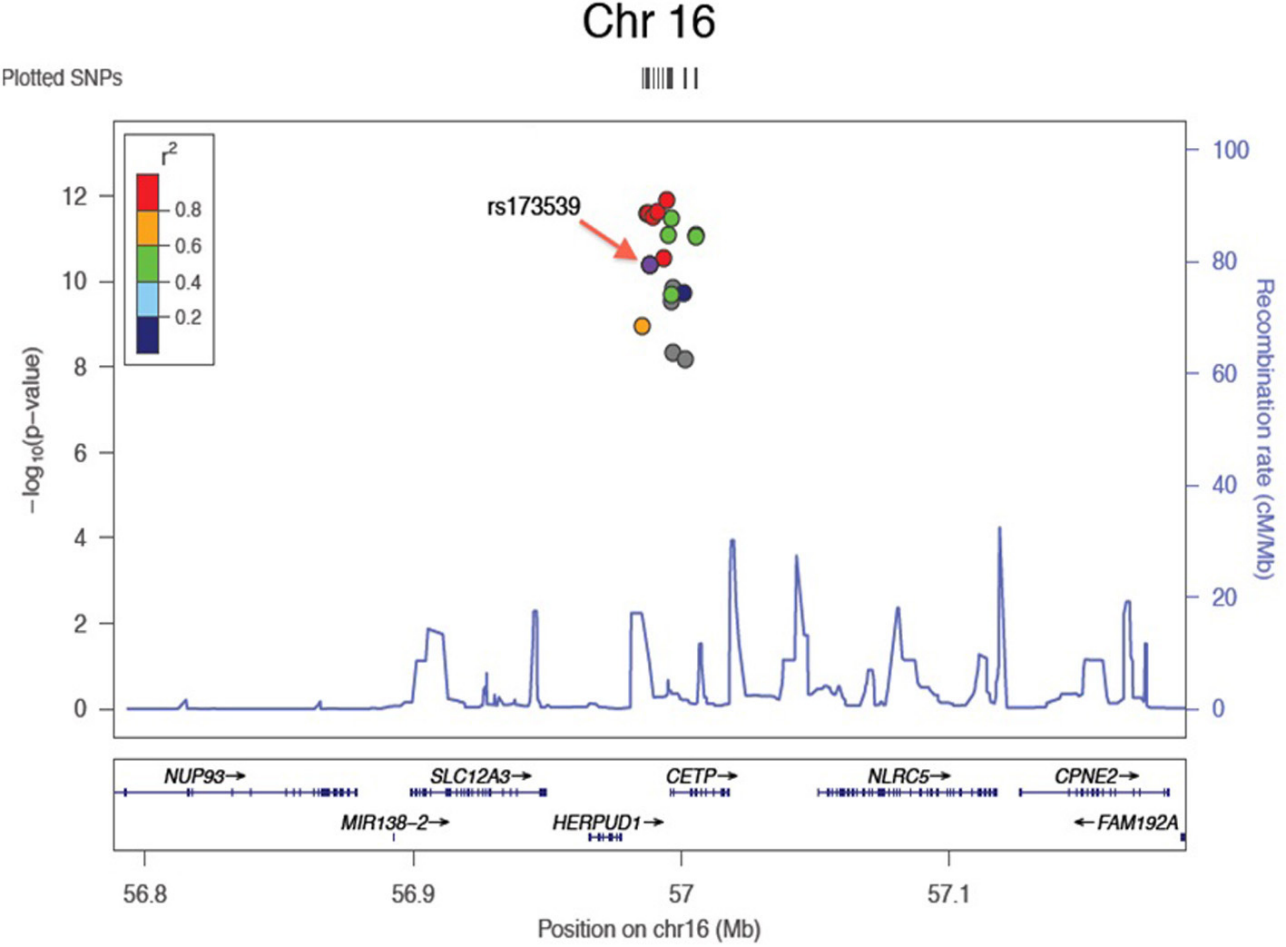
**Zoom plot showing association of SNPs in CETP region (Chromosome 16) associated with HDL-C, and linkage disequilibrium between associated SNPs and index SNP**.

**Figure 3:**
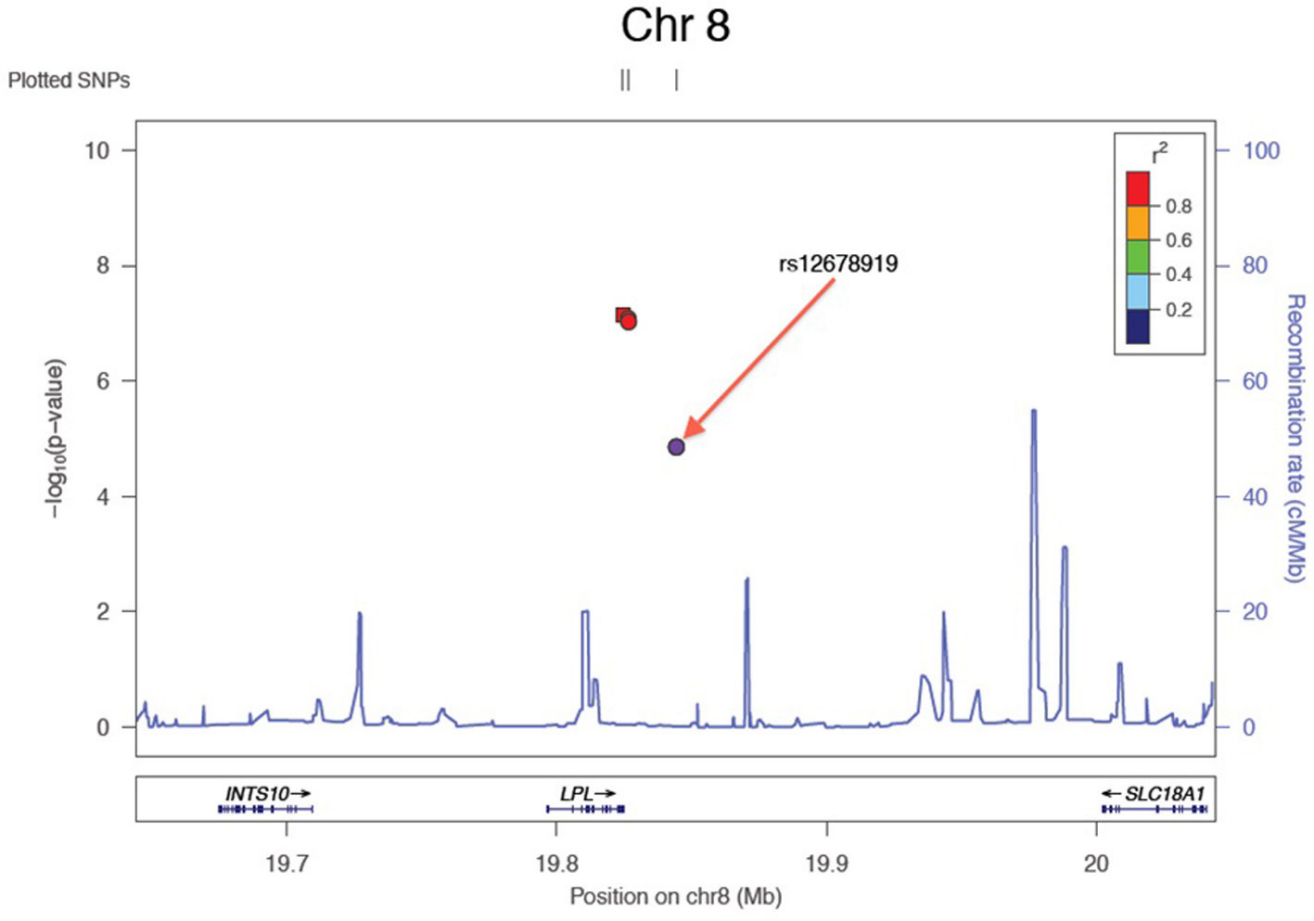
**Zoom plot showing association of SNPs in LPL region (Chromosome 8) associated with HDL-C, and linkage disequilibrium between associated SNPs and index SNP**.

Results of the array-wide association analyses for 5 lipid phenotypes are summarized in Manhattan plots shown in Figures 1A–E. SNPs at three loci achieved *p*-values at less than 1 × 10^−7^ in association with HDL-C (Figure 1A). A cluster of SNPs with *p*-values less than 1 × 10^−12^ was identified at the *HERPUD1-CETP* locus on chromosome 16 (Keebler et al., 2009). All associated SNPs were in high linkage disequilibrium with rs173539 (Figure 2), the peak SNP identified in this region previously associated with HDL-C (Kathiresan et al., 2008). A cluster of SNPs at the *LPL* locus on chromosome 8 also associated with HDL-C levels, as has been previously reported (Heid et al., 2008). As shown in Figure 3, the associated SNPs were in high linkage disequilibrium with rs12678919, the peak SNP previously identified in this region associated with HDL-C (Kathiresan et al., 2008). The third locus associated with HDL-C levels was tagged by only a single SNP with a *p*-value of 7.46 × 10^−9^ was located at the *NPAS3* locus.

For TC (Figure 1B), the peak association occurred with multiple SNPs on chromosome 1 at the *CELSR2-PSRC1-SORT1* locus (peak association: *p* < 2.6 × 10^−7^), which has previously been associated with TC in multiple studies (Lu et al., 2010; Ma et al., 2010). Associations at this locus were also apparent for LDL-C levels (Figure 1C), as has been reported in previous studies (Kathiresan et al., 2008; Nakayama et al., 2009), although the associations did not achieve the Bonferroni-corrected level of statistical significance. The well-documented association of LDL-C levels with *APOE* on chromosome 19 (Waterworth et al., 2010), was also detected, with a *p*-value of 1.56 × 10^−12^.

A significant association was observed between TG and the *APOA1*-*APOA3*-*APOA4*-*APOA5* locus on chromosome 11 (SNP rs number not available; resides at position 116,156,325; see Figure 1D) as has been previously reported (Johansen et al., 2011). Statistically significant associations with TC/HDL-C ratio were found at three loci (Figure 1E), two overlapping with TC and LDL-C, the *CELSR2-PSRC1-SORT1* and *APOE* regions.

## Discussion

Large-scale genomic studies can be costly due to the large sample sizes required for sufficient power to detect statistically significant differences of alleles with small to moderate effect sizes. Both phenotyping and genotyping can be expensive depending upon what is required for phenotyping and which genotype platform is selected. For this study, we used phenotype data derived from a clinical database constructed using EHR data (Wood et al., 2012). The laboratory data were obtained as part of clinical standard of care saving on the costs of thousands of blood lipid analyses; the anthropomorphic and demographic data were obtained by professionally certified clinicians and providers remunerated as part of clinical care. Importantly, the data were obtained in an electronic format allowing for the efficient construction of a flexible database. Despite the electronic format, careful data quality control and scrubbing were required in order to ensure robust results. In particular, the recording of body weights in a cohort of patients with extreme obesity must be carefully curated to ensure accuracy. Similarly, despite selection for high success rates, generating accurate SNP calls for the Metabochip platform required quite extensive manual curation and data cleaning.

We successfully extended to an extremely obese cohort associations between SNPs identified by large meta-GWAS studies and lipid traits. Failure to replicate associations with many of the SNPs was likely due to insufficient power from the smaller sample size in our cohort vis-à-vis the very large populations analyzed in previous studies. Alternatively, it is possible that some of the non-replicated SNPs have smaller effect sizes in extreme obese populations. However, the direction of effects and the effect sizes were similar to that previously reported by the large meta GWAS studies in all but two of the SNPs analyzed.

The SNPs we associated with TC and LDL-C largely overlapped. This is not surprising since the value for LDL-C is calculated using the Friedewald equation based on adjusting TC for HDL-C and TGs levels (subtract HDL-C and one-fifth of the TGs from TC). They are thus correlated values, so linear regression analyses will identify similar associations. Medication use was also not associated with either TC or LDL-C. Levels of HDL-C appear to be under strong genetic control, yet despite such high heritability (Ober et al., 2006), GWAS loci do not explain a large proportion of HDL-C variation (Willer and Mohlke, 2012). Nevertheless, we replicated several known loci. One locus, *LPL*, has also been robustly associated with risk for cardiovascular disease (Deloukas et al., 2013). Our results indicate that this locus may therefore also be a risk locus for CVD in patients with extreme obesity similar to previous studies of other CVD loci (Wood et al., 2008). Our results for HDL-C are similar to those reported for a bariatric surgery cohort of similar sample size in which a total of 60 SNPs in the ATP-binding cassette, sub-family A member 1 (*ABCA1*), apolipoprotein A1/C3/A4/A5 cluster (*APOA5*), cholesterol ester transfer protein (*CETP*), UDP-GalNAc transferase 2 (*GALNT2*), hepatic lipase (*LIPC*), endothelial lipase (*LIPG*), lipoprotein lipase (*LPL*), and the methylmalonic aciduria cblB type (*MMAB*)/mevalonate kinase (*MVK*) loci were genotyped (Sarzynski et al., 2011). Only SNPs in the *LPL*, *LIPC*, and *CETP* loci were statistically associated with pre-operative HDL-C level, similar to our results, although the multiple test correction factor was far less stringent than ours.

We found only a single locus associated with TG that replicated from the loci reported by the Global Lipids Genetics Consortium meta-analysis of over 100,000 individuals comprised of multi-ethnic and multi-racial populations (Teslovich et al., 2010). This is likely due to the much smaller sample size or alternatively, variation at the *APOA1/C3/A4/A5* gene cluster (Lai et al., 2005) may be the only genetic locus of the previously identified loci that associates with TG in extreme obesity. Which gene or genes in the *APOA1/C3/A4/A5* cluster harbors the TG influencing variant is not known.

A limitation of our study is that many of our subjects were on lipid lowering medications that may have masked our ability to identify genetic associations. A priori, lowering (or raising in the case of HDL-C) lipid levels through medication use may be expected to disproportionately occur in subjects with dyslipidemia due to a genetic predisposition, thus decreasing the ability to identify lipid-genotype associations. To address this issue, we performed three complementary analyses, including removing all subjects on lipid-lowering medications, the strategy employed by large meta-GWAS (Kathiresan et al., 2008). This predictably led to a major loss of statistical power, perhaps acceptable for very large sample sizes but not for our cohort. The approach of adjusting for medication use as a covariate had virtually no effect on the sensitivity of detecting SNP-lipid associations. However, this approach is biased by the indication for medication being high lipid levels, thus any identified associations between genotype and lipid levels are not independent of medication use.

Another potential limitation is that some patients may not have been fasting for a sufficient length of time prior to the blood draw to avoid an artifactual dietary effect on blood lipid measurements. For example, triglyceride levels are particularly sensitive to prandial state and other influences (Yuan et al., 2007). About 25 patients had triglyceride levels greater than 500 mg/dl, considered the highest category of hypertriglyceridemia by the Adult Treatment Panel III of the National Cholesterol Education Program (Expert Panel on Detection, Evaluation, and Treatment of High Blood Cholesterol in Adults, 2001). Hypertriglyceridemia may be expected to have a higher prevalence in populations with extreme obesity. In addition, assuming that the probability of non-fasting was independent of genotype, one would expect that measurement error due to non-fasting would obscure gene-lipid associations, not create false positives. The replication of associations at known loci supports the utility of using clinical samples.

No SNPs were found to be significantly associated with either BMI or waist circumference. The SNPs selected for the Metabochip included those from GWAS from the Genetic Investigation of Anthropometric Traits (GIANT) consortium, which studied anthropometric traits BMI and waist circumference. A total of 18,211 SNPs from 24 loci (Expert Panel on Detection, Evaluation, and Treatment of High Blood Cholesterol in Adults, 2001; Brahm and Hegele, 2013) found to be associated with BMI, plus 5055 replication SNPs, were included on the Metabochip, along with 1374 SNPs and 1048 replication SNPs from 2 loci associated with waist circumference (Yuan et al., 2007). These studies involved multiple cohorts each with a mean BMI of about 27–28 kg/m^2^ with a standard deviation of less than 5 kg/m^2^. The proportion of these cohorts with a BMI of greater 40 kg/m^2^ is thus likely less than 5–10%, with a relatively limited range of BMIs of less than 25 kg/m^2^. The cohort with extreme obesity studied here had an average BMI of 48 ± 8 kg/m^2^, representing a much higher average BMI, as well as a much wider range in BMI. The BMI of individuals at the upper range of the human body weight distribution may represent a distinct phenotype (Still et al., 2011) and harbor rarer variants with higher penetrance and larger effect sizes than the common variants interrogated by the Metabochip platform. Next generation sequencing may be required to identify those variants (Gerhard et al., 2013).

In summary, we conducted a GWAS of major lipid traits using EHR derived data to analyze SNPs that had previously been associated with lipid phenotypes, as well as other SNPs residing on the Metabochip, in an extremely obese cohort. Although several lipid loci replicated, other previously identified lipid and body weight loci did not. Possible differences may be due to the use of EHR data for phenotyping, characteristics of the cohort, and/or decreased statistical power. Nevertheless, the availability of extant EHR phenotype data and the relatively low cost of the specialized Metabochip can be effectively used to conduct a GWAS.

### Conflict of interest statement

The authors declare that the research was conducted in the absence of any commercial or financial relationships that could be construed as a potential conflict of interest.
