# Supplementary material for: Extension of GWAS results for lipid-related phenotypes to extreme obesity using electronic health record (EHR) data and the Metabochip
Source: Front Genet. 2014 Aug 5;5:222. doi: 10.3389/fgene.2014.00222 (PMC4123014; doi:10.3389/fgene.2014.00222)
Supplement: Supplementary file 1 [file DataSheet1.PDF]

Extension of GWAS results for lipid-related phenotypes to extreme obesity using electronic health record (EHR) data and the Metabochip.

Parihar, et. al.

Supplementary Information

Supplementary Tables

Supplementary Table 1. Flow chart of sample QC with exclusions

Supplementary Table 2. Frequency of diagnoses and medication use.

Supplementary Table 3. Adjustment of lipid levels in subjects taking lipid-lowering medications.

Supplementary Table 4. GWAS results compensating for lipid medication use.

Supplementary Table 5. Associations of SNPs at known body weight loci with BMI and waist circumference.

Supplementary Figures

Supplementary Figure 1. Manhattan plot of BMI.

Supplementary Figure 2. Manhattan plot of waist circumference.

Supplementary Figure 3. Manhattan plots of lipid levels excluding subjects taking lipid-lowering medications.

Supplementary Figure 4. Manhattan plots of lipid levels including medication use as a covariate.

Supplementary Figure 5. Manhattan plots of lipid levels including only subjects taking lipid-lowering medications.

Supplementary Methods

Supplementary Table 1. Flow chart of sample QC with exclusions.

Filters for QC	Excluded samples	Number of samples
**Total sample number genotyped**		1851
(Call rates < 0.95)	24	
(Missing phenotypes)	8	
**Genotyped and phenotyped samples with acceptable call rate**		1819
(Unresolved gender discrepancy)	25	
(Unexpected duplicate)	30	
(Improbable parent-offspring pair based on age difference)	6	
**Total 'cleaned' sample set**		1758
Additional exclusions for primary analyses:		
(Parent-offspring pairs, keep offspring only)	41	
(Sibling pair and half sibling pair, keep youngest sib only)	38	
Sample ids occurring in multiple pairs	+7	
**Final analysis set** (1670 Caucasian; 16 non-Cauc by PC analysis)		1686

Supplementary Table 2. Frequency of diagnoses and medication use.

Medication	Percent
Diagnosis of Hypertension	48.7
Diagnosis of Type 2 Diabetes	35.2
Biguanides	41.1
Insulin	14.6
Sulfonylureas	12.5
Insulin Sensitizing Agents	12.1
Incretin Mimetic Agents	2.3
Diabetes Combo Meds	1.9
Dpp4 Inhibitors	1.8
Statins	34
Fibric acid derivatives	6.4
Bile acid sequestrants	0.2
Nicotinic acid derivatives	0.5
Combination therapy	4.6
Any lipid-lowering medication	45.7

Supplementary Table 3. Adjustment of lipid levels in subjects taking lid-lowering medications.

Lipid Lowering Medication	Percentage of Population	LDL-C Adjustment (Reference)	TCHOL Adjustment (Reference)*	HDL-C Adjustment (Reference)	TGs Adjustment (Reference)
Statins	34%	↓ 38% (2)	=LDL+HDL +(TG/5)	↑ 7% (1)	↓ by 10% (6)
Fibric acid derivatives	6%	↓ by 12% (8)	=LDL+HDL +(TG/5)	↑ by 10% (1)	↓ by 40% (8)
Bile acid sequestrants	1%	↓ 13% (7)	=LDL+HDL +(TG/5)	↑ by 4% (5)	↓ 0% (5)
Nicotinic acid derivatives	1%	↓ by 15% (4)	=LDL+HDL +(TG/5)	↑ by 25% (4)	↓ by 35% (4)
Combination Therapy (statins with fibrates)	4%	↓ by 40% (3)	=LDL+HDL +(TG/5)	↑ by 20% (3)	↓ by 50% (3)

* adjusted total cholesterol levels obtained by Friedenwald equation, based on the adjusted levels for LDL-C, HDL-C, and TGs.

Equations used to calculate adjusted lipid levels:

Statin users:

Adj LDL = LDL / (1 - 0.38)

Adj HDL = HDL / (1 +0.07)

Adj TG = TG / (1 – 0.10)

Adj TCHOL = AdjLDL + AdjHDL + (AdgTG/5)

Fibric acid derivatives users:

Adj LDL = LDL / (1 - 0.12)

Adj HDL = HDL / (1 +0.10)

Adj TG = TG / (1 – 0.40)

Adj TCHOL = AdjLDL + AdjHDL + (AdgTG/5)

Bile acid sequestrants users:

Adj LDL = LDL / (1 - 0.13)

Adj HDL = HDL / (1 +0.04)

Adj TG = TG / (1)

Adj TCHOL = AdjLDL + AdjHDL + (AdgTG/5)

Combination therapy users:

Adj LDL = LDL / (1 - 0.15)

Adj HDL = HDL / (1 +0.25)

Adj TG = TG / (1 – 0.35)

Adj TCHOL = AdjLDL + AdjHDL + (AdgTG/5)

Fibric acid derivatives users:

Adj LDL = LDL / (1 - 0.40)

Adj HDL = HDL / (1 +0.20)

Adj TG = TG / (1 – 0.50)

Adj TCHOL = AdjLDL + AdjHDL + (AdgTG/5)

References:

1. Meyers CD, Kashyap ML. Pharmacologic augmentation of highdensity lipoproteins: mechanisms of currently available and emerging therapies. Curr Opin Cardiol. 2005;20(4):307-312.

2. Naci, Huseyin, et al. "Dose-comparative effects of different statins on serum lipid levels: a network meta-analysis of 256,827 individuals in 181 randomized controlled trials." *European journal of preventive cardiology* 20.4 (2013): 658-670.

3. Saseen, Joseph J., and Elizabeth M. Tweed. "What are effective medication combinations for dyslipidemia?." *Clinical Inquiries, 2006 (MU)* (2006).

4. Boden, William E., Mandeep S. Sidhu, and Peter P. Toth. "The Therapeutic Role of Niacin in Dyslipidemia Management." *Journal of cardiovascular pharmacology and therapeutics* (2013): 1074248413514481.

5. Ascaso JF. Advances in cholesterol-lowering interventions. *Endocrinol. Nutr.*57(5),210–219 (2010).

6. Scandinavian Simvastatin Survival Study Group. Randomised trial of cholesterol lowering in 4444 patients with coronary heart disease: the Scandinavian Simvastatin Survival Study (4S). Lancet. 1994;344:1383-1389. Abstract

7. The Lipid Research Clinics Coronary Primary Prevention Trial results. II. The relationship of reduction in incidence of coronary heart disease to cholesterol lowering [no authors listed]. JAMA. 1984; 251:365–374.
8. Khoury, N., and Goldberg, A.C. (2011). The use of fibric Acid derivatives in cardiovascular prevention. Curr Treat Options Cardiovasc Med 13, 335-342.

Supplementary Table 4. GWAS results ($p < 1 \times 10^{-5}$) compensating for lipid medication use.

					All Subjects (n = 1686)		Exclude subjects taking lipid-lowering medications (n = 945)		Include medication use as a covariate (n = 1686)		Include ONLY subjects taking lipid-lowering medications (n = 741)	
Trait	Peak SNP	Locus	CHR	CHR Position (HG18)	Beta †	P-value	Beta †	P-value	Beta †	P-value	Beta †	P-value
HDL	rs10801892		1	88,681,806	1.02	1.60 E-02	3.55	1.14 E-06	1.06	1.13 E-02	-0.63	1.14 E-01
Chol	chr1:109619829	SORT1	1	109,619,829	-8.37	3.03 E-07	-7.29	8.66 E-04	-0.23	2.88 E-06	-6.44	2.84 E-02
LDL	chr1:109619829	SORT1	1	109,619,829	-7.10	3.96 E-07	-6.73	3.83 E-04	-7.20	2.66 E-07	-4.27	8.47 E-02
Chol	chr1:109623666	SORT1	1	109,623,666	-8.38	2.49 E-07	-7.59	4.93 E-04	-0.23	1.35 E-06	-6.28	3.21 E-02
LDL	chr1:109623666	SORT1	1	109,623,666	-7.01	4.80 E-07	-6.85	2.76 E-04	-7.12	3.19 E-07	-4.05	1.02 E-01
Chol	chr2:514173		2	514,173	-9.22	2.27 E-04	-15.51	7.73 E-06	10.68	0.0000021 9	-7.75	9.27 E-02
Chol	chr6:20603525	E2F3	6	20,603,525	5.98	6.23 E-04	2.12	3.73 E-01	0.24	4.21 E-06	4.97	1.19 E-01
Chol	rs628751		6	139,880,112	2.59	6.18 E-02	-1.35	4.69 E-01	0.19	4.09 E-06	7.44	3.14 E-03
Chol	rs4339520	WBSCR17	7	70,293,877	9.32	3.49 E-04	9.95	5.54 E-03	0.39	4.72 E-07	-0.34	9.44 E-01
HDL	chr8:19868843	LPL	8	19,868,843	3.22	7.34 E-08	3.00	2.42 E-03	3.16	9.97 E-08	0.97	3.22 E-01
TRIG	rs6537571		10	49,556,819	-0.08	4.25 E-06	-0.08	2.64 E-03	-0.08	6.00 E-06	-0.03	1.16 E-02
Trig	chr11:8468011	STK33	11	8,468,011	0.01	7.27 E-01	-0.01	7.85 E-01	0.01	6.98 E-01	0.04	8.83 E-03

TG	chr11:116156325	APOA1 APOA3 APOA4 APOA5	11	116,156,325	0.11	3.31 E-08	0.11	4.67 E-04	0.11	3.56 E-08	1.37	8.71 E-02
HDL	rs10131323	NPAS3	14	33,009,820	3.25	7.46 E-09	4.48	8.89 E-06	3.25	5.72 E-09	1.08	2.91 E-01
HDL	rs247617	CETP	16	55,548,217	2.88	1.36 E-12	3.26	1.63 E-08	7.20	9.344 E-13	6.01	9.22 E-02
Chol	rs2075650	TOMM40	19	50,087,459	9.37	4.05 E-06	8.71	1.97 E-03	0.32	1.07 E-07	3.92	1.92 E-01
LDL	rs2075650	TOMM40	19	50,087,459	8.98	2.41 E-07	9.89	4.89 E-05	9.12	1.60 E-07	5.49	2.34 E-01
Chol	chr19:50103919	APOE	19	50,103,919	-8.31	7.05 E-04	-13.70	1.88 E-05	-0.34	2.51 E-06	-3.69	3.40 E-01
LDL	chr19:50103919	APOE	19	50,103,919	-14.72	1.56 E-12	-19.06	3.77 E-12	-15.15	4.19 E-13	-0.63	1.14 E-01

† effect on lipid levels (expressed in SD units) associated with each copy of the reference allele

Table 5. Associations of SNPs at known body weight loci with BMI and waist circumference.*

						From (Speliotes et al., 2010)			Association results		
Trait	SNP	CHR	POS (HG18)	Gene	Reference Allele	Allele Frequency	Beta†	P-value	Allele Frequency	Beta †	P-value
BMI	rs2815752	1	72,585,028	NEGR1	G	0.61	0.13	1.61E−22	0.65	-0.75	0.01
BMI	rs1514175	1	74,764,232	TNNI3K	A	0.43	0.07	8.16E−14	0.45	-0.31	0.28
Waist	rs543874	1	176,156,103	SEC16B	G	0.19	0.22	3.56E−23	0.21	0.35	0.18
BMI	rs2867125	2	612,827	TMEM18	A	0.83	0.31	2.77E−49	0.84	-0.41	0.30
BMI	rs887912	2	59,156,381	FANCL	A	0.29	0.10	1.79E−12	0.30	0.14	0.65
BMI	rs13078807	3	85,966,840	CADM2	G	0.20	0.10	3.94E−11	0.21	0.44	0.20
BMI	rs10938397	4	44,877,284	GNPDA2	G	0.43	0.18	3.78E−31	0.48	0.29	0.28
BMI	rs13107325	4	103407,732	SLC39A8	A	0.07	0.19	1.50E−13	0.09	0.11	0.82
BMI	rs2112347	5	75,050,998	FLJ35779, HMGCR	C	0.63	0.10	2.17E−13	0.66	-0.23	0.45
BMI	rs206936	6	34,410,847	NUDT3	G	0.21	0.06	3.02E-08	0.21	0.47	0.17
Waist	rs987237	6	50,911,009	TFAP2B	G	0.18	0.13	2.90E−20	0.22	0.01	0.97
BMI	rs10968576	9	28,404,339	LRRN6C	G	0.31	0.11	2.65E−13	0.33	0.09	0.75
BMI	rs3817334	11	47,607,569	MTCH2	A	0.41	0.06	1.59E−12	0.41	-0.37	0.19
Waist	rs7138803	12	48,533,735	FAIM2	A	0.38	0.12	1.82E−17	0.38	0.60	0.01
BMI	rs2241423	15	65,873,892	MAP2K5	A	0.78	0.13	1.19E−18	0.78	-0.09	0.78
BMI	rs12444979	16	19,841,101	GPRC5B	A	0.87	0.17	2.91E−21	0.87	-1.09	0.01
BMI	rs7359397	16	28,793,160	SH2B1	A	0.40	0.15	1.88E−20	0.41	0.46	0.10
BMI	rs571312	18	55,990,749	MC4R	A	0.24	0.23	6.43E−42	0.26	0.04	0.89
BMI	rs29941	19	39,001,372	KCTD15	A	0.67	0.06	3.01E−09	0.69	-0.48	0.12
BMI	rs2287019	19	50,894,012	GIPR	A	0.80	0.15	1.88E−16	0.79	0.40	0.25
BMI	rs3810291	19	52,260,843	TMEM160	G	0.67	0.09	1.64E−12	0.68	+	0.30

* SNPs at known BMI and waist-associated loci taken from (Speliotes et al., 2010).

† effect sizes per unit BMI or waist circumference (expressed in SEM units) associated with each copy of the reference allele

Supplementary Figure 1. Manhattan plot of BMI.

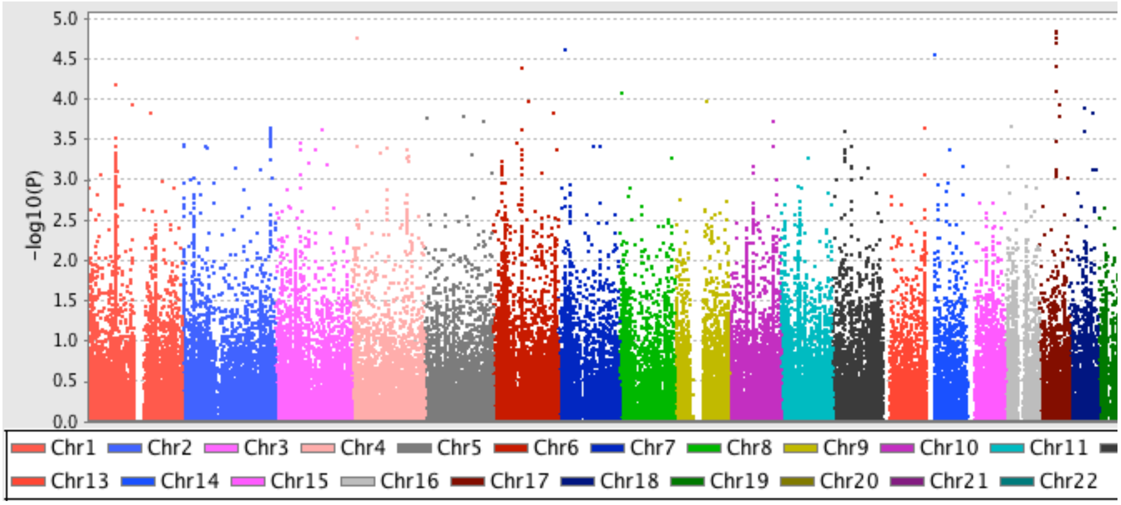

Supplementary Figure 2. Manhattan plot of waist circumference.

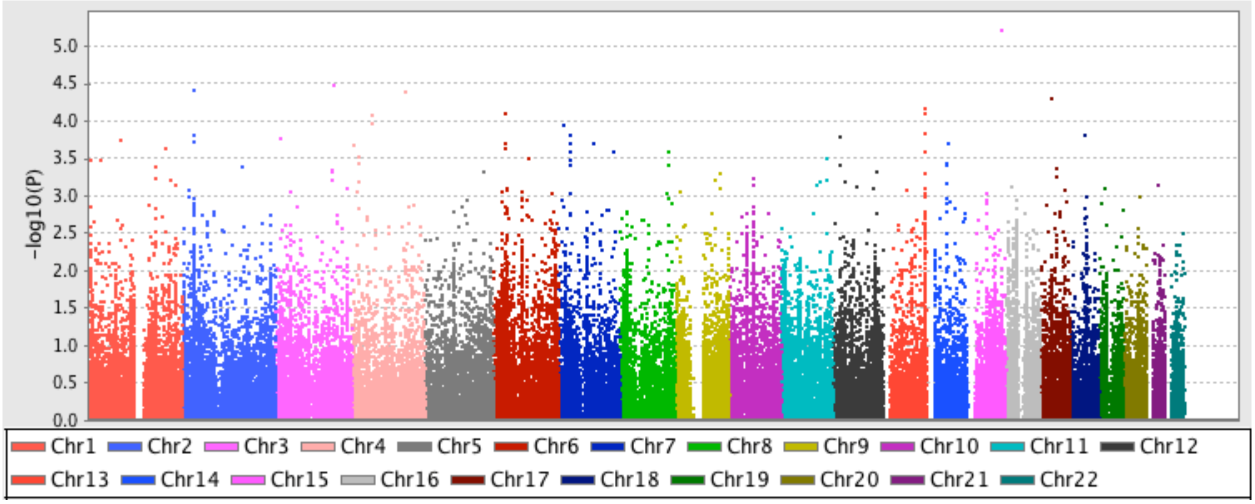

Supplementary Figure 3. Manhattan plots of lipid levels EXCLUDING subjects taking lipid-lowering medications.

Supplementary Figure 3a. HDL-C

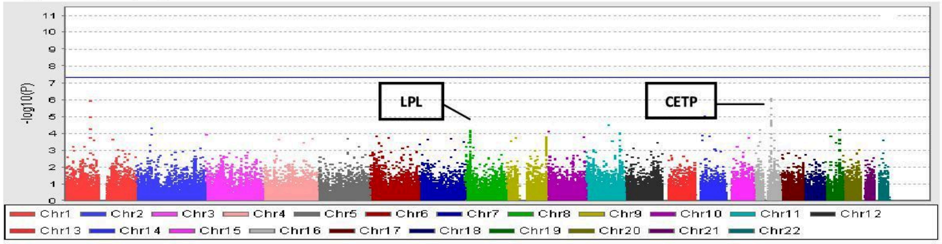

Supplementary Figure 3b. TC

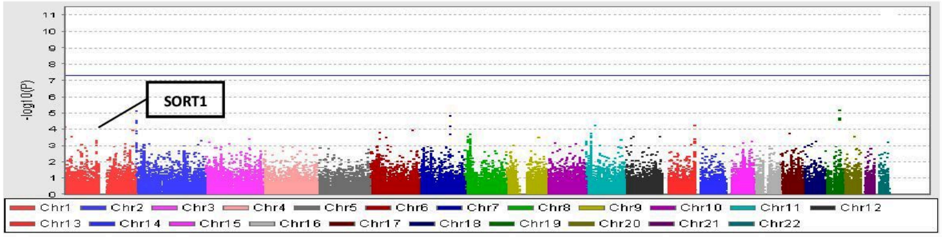

Supplementary Figure 3c. TC/HDL-C

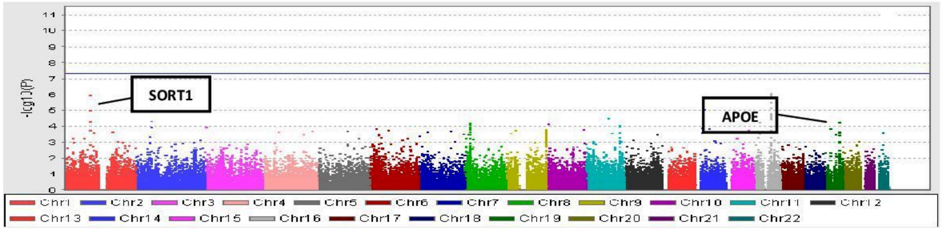

Supplementary Figure 3d. TG

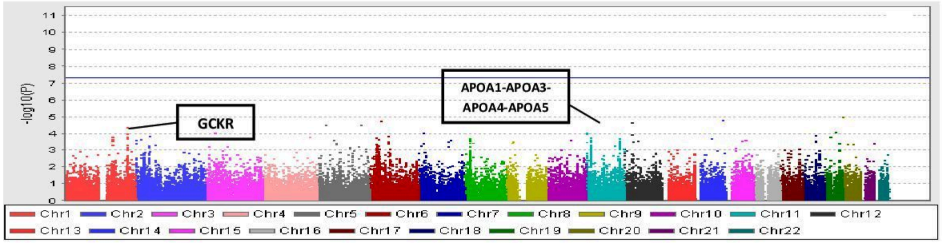

Supplementary Figure 3e. LDL-C

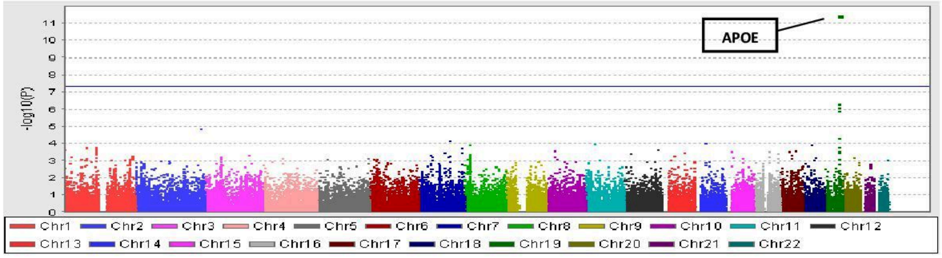

Supplementary Figure 4. Manhattan plots of lipid levels including medication use as a covariate.

Supplementary Figure 4a. HDL-C

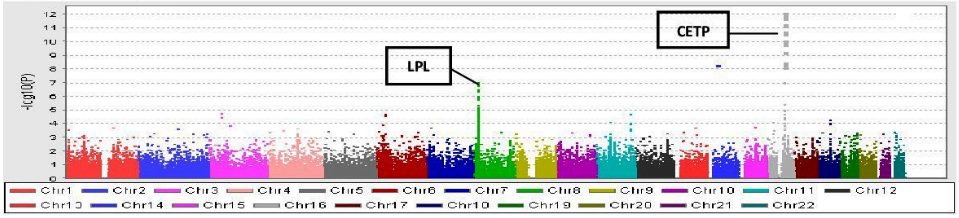

Supplementary Figure 4b. TC

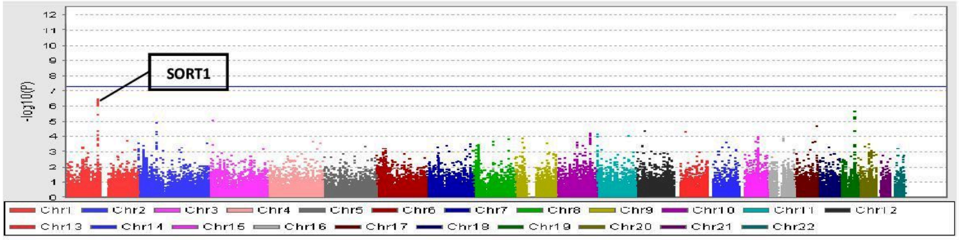

Supplementary Figure 4c. TC/HDL-C

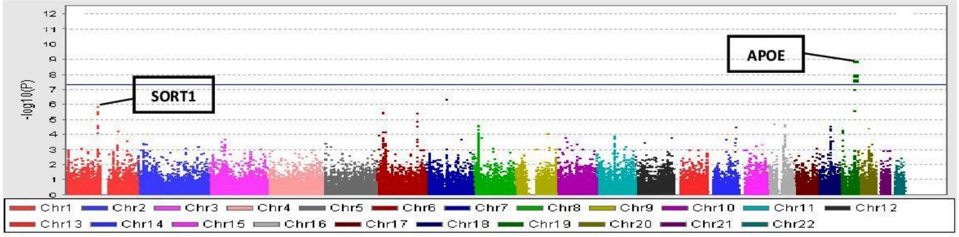

Supplementary Figure 4d. TG

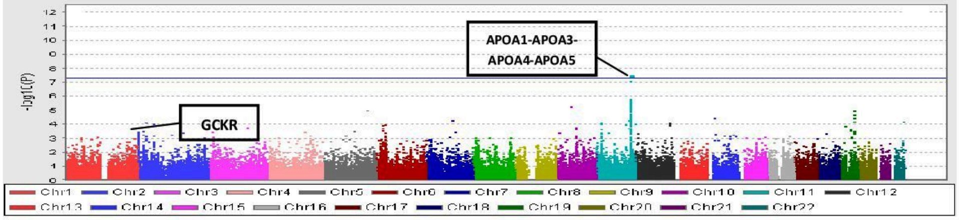

Supplementary Figure 4e. LDL-C

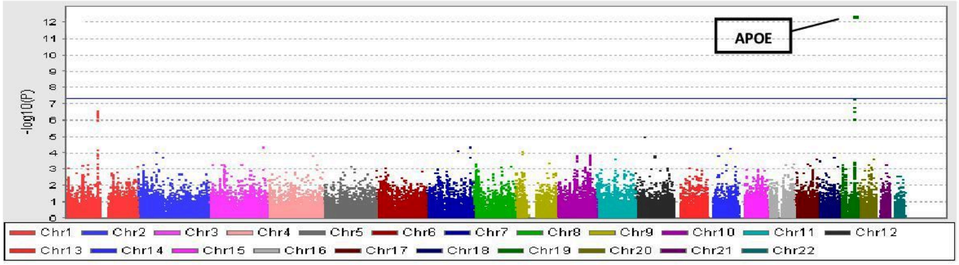

Supplementary Figure 5. Manhattan plots of lipid levels INCLUDING ONLY subjects taking lipid-lowering medications.

Supplementary Figure 5a. HDL-C

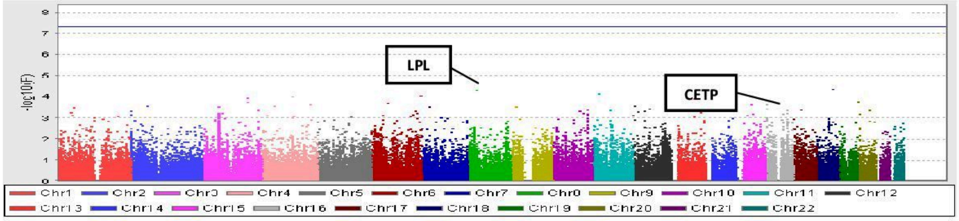

Supplementary Figure 5b. TC

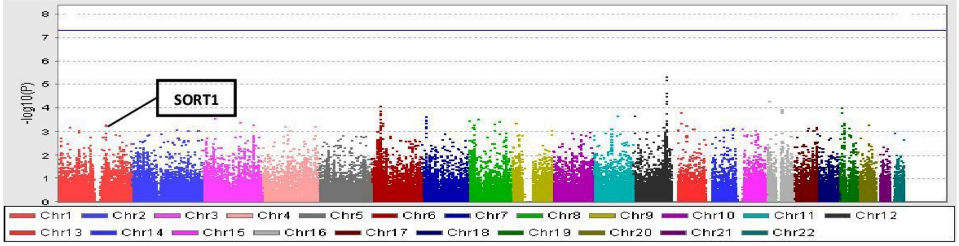

Supplementary Figure 5c. TC/HDL-C

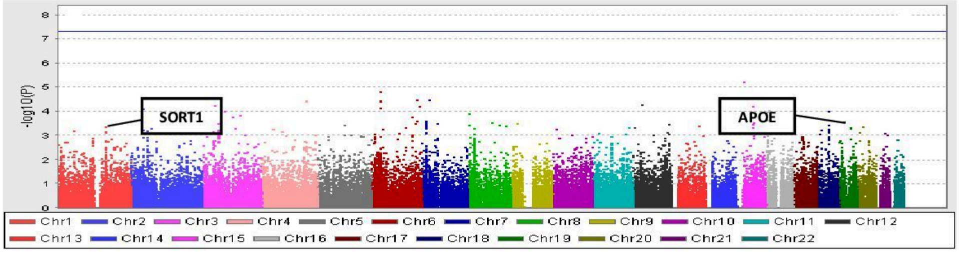

Supplementary Figure 5d. TG

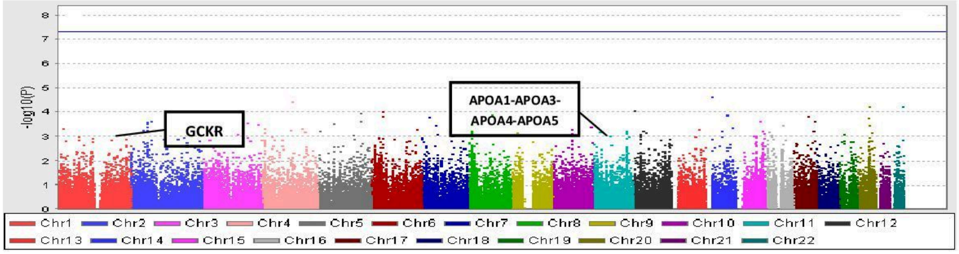

Supplementary Figure 5e. LDL-C

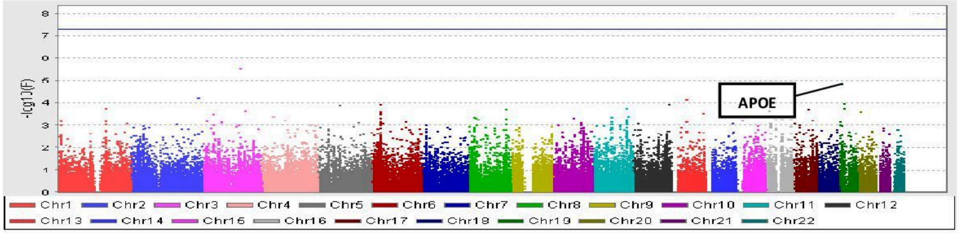

Supplementary Methods

Sample QC

We evaluated genotyping results to identify potentially problematic samples suggestive of poor quality DNA and/or gender mismatches. To verify consistency of the annotated (self-reported) gender and gender assignment as determined by genetic analysis, we evaluated heterozygosity of the samples based on X and Y chromosome SNPs and used the 'estimate gender' function from Illumina's Genome Studio. The self-reported gender of 25 samples (14 reported male and 11 female) did not match the estimated gender of samples based on genotype data and were removed from the sample set.

We next evaluated the relatedness between each pair of samples by estimating probabilities of allele sharing using the Identity-by-descent (IBD) option as implemented in PLINK (Purcell et al., 2007). For these analyses we included a subset of 104,714 MetaboChip SNPs that were LD-pruned (at $r^2 < 0.1$), and had mean allele frequency (MAF) > 0.05. We plotted the IBD coefficients Z0 and Z1 using R (Supplement Figure 1), where Z0 and Z1 represent for each relative pair the estimated probabilities of sharing 0 and 1 alleles IBD across all SNPs. From these plots, we identified 101 pairs with close relationship types, including 16 duplicate pairs (100% probability of sharing 2 alleles), 44 parent-child pairs (100% probability sharing 1 and only 1 allele), 25 pairs of siblings (25%, 50%, and 25% probabilities of sharing 0, 1, and 2 alleles, respectively), and 16 pairs of half-siblings (50% probability of sharing 0 alleles and 50% probability of sharing 1 allele). For 3 of the 16 duplicate samples, one sample from each pair was selected for inclusion by assigning the DNA sample to the sex-matched phenotype

record; the remaining samples were excluded. For parent-child, sibling, and half-sib pairs, only 1 individual from each pair (the younger) was included for association analysis.

SNP QC

We initially identified a total of 5,968 problematic SNPs based on low call rates (< 95%) and ambiguity in establishing clear genotype clusters (Supplementary Methods). The exclusions were based on quality control thresholds suggested by the Illumina FastTrack Genotyping Services Group for clustering, calling, scoring, and analyzing genotype data. These suggested filter criteria were then adjusted based on visual inspection and manual re-clustering of autosomal SNPs because ~20,000 good performing SNPs were being excluded by the relatively stringent thresholds. To identify additional SNPs potentially manifesting poor clusters, we performed an analysis of all remaining SNPs to evaluate deviation from HW expectations. These analyses, performed only on SNPs with MAF greater than 5%, identified 1,196 SNPs that showed significant evidence for deviation from HWE at a p-value threshold of $p < 1 \times 10^{-5}$. We manually analyzed the clusters of these SNPs and classified 311 of them as poor performing SNPs due to unacceptable clusters and removed them from the dataset. The remaining 885 SNPs deviating from HW equilibrium had acceptable clusters and were included in the final set. As a final level of quality control, the cluster plots of any SNPs found to be in association with a phenotype on final analysis were also visually inspected.
